# The Impact of Silver Diamine Fluoride and Potassium Iodide Treatment on Microtensile Bonds Strength of Composite Restoration for Carious Dentin in Primary and Permanent Teeth and Its Potential Color Changes: An *In Vitro* Study

**DOI:** 10.1055/s-0044-1791684

**Published:** 2024-11-21

**Authors:** Sarah D. Shaheen, Doaa A. M. Esmaeil, Somaia Ghobar

**Affiliations:** 1 Conservative surgery Department, Collage of Oral and Dental Surgery, Misr University for Science and Technology (MUST), Giza, Egypt; 2 Oral Pathology Department, Faculty of Dentistry, Mansoura University, Mansoura, Egypt; 3 Oral Pathology Department, Faculty of Dentistry, Sinai University, Kantara Campus, Ismailia, Egypt; 4 Pediatric Dentistry & Public Health, Faculty of Dentistry, Sinai University, Kantara Campus, Ismailia, Egypt

**Keywords:** silver diamine fluoride (SDF), potassium iodide (KI), tensile strength

## Abstract

**Objectives** This study aims to examine the effects of silver diamine fluoride and potassium iodide (SDF/KI) treatment on the possible color changes and the microtensile bond strength of composite restorations to carious dentin in different dentitions.

**Material and Methods** A total of 48 sound human teeth were utilized in this study. Twenty-four primary molar teeth were divided into two groups. Each group has 12 teeth; group 1 received no pretreatment, while group 2 received SDF/KI treatment. Also, 24 permanent premolar teeth were divided into two groups. Group 3 received no pretreatment, and group 4 received SDF/KI treatment. For 7 days at 25 °C, every sample was submerged in a demineralizing solution. Following the manufacturer's guidelines for SDF/KI treatments, exposed dentin surfaces were promptly preserved in artificial saliva (pH 7.4) for 14 days. On the prepared teeth surfaces of the nontreated groups, deionized water was administered rather than SDF/KI. A self-etching bonding agent was used, and a 4-mm-thick composite restoration was constructed. Evaluations were conducted on color measurement, microtensile bond strength, and mode of failure.

**Statistical Analysis** The groups were compared using a one-way analysis of variance test, and a post hoc test was used for pairwise comparisons.

**Result** Regarding color measurement, in deciduous dentition, there were no statistically significant differences between the groups. Additionally, there is no discernible variation in Δ *E* values among various pretreatment groups in the permanent dentition. Microtensile bond strength did not differ significantly between permanent and deciduous teeth.

**Conclusion** A 14-day delay in the application of composite restoration can successfully conceal the color of SDF/KI without compromising the microtensile bond strength.

## Introduction

Silver diamine fluoride (SDF) is a cost-effective caries-prevention agent. It is among the preventive agents commonly used in minimally invasive dentistry. It has been authorized for use as a therapeutic medication to arrest caries. SDF possesses antimicrobial properties and remineralizing ability of dental tissue. 1 2

According to multiple laboratory investigations, SDF produces a dark silver precipitate of Ag(s) that discolors dentin in addition to the free fluoride ions and CaF2 required to remineralize dental tissues. 3 4

However, one of the main drawbacks of applying SDF alone to tooth structure is discoloration. As previously documented, sound tooth structure and carious tissues are discolored dark brown or black following SDF. Silver ion staining increased with the amount of demineralized region. The stain persists over time and can only be removed by physical means. 5

The combination of SDF and potassium iodide (KI) was proposed as a discoloration treatment. As KI minimizes the discoloration caused by SDF without affecting its effectiveness in preventing caries. 6

Another proposal to solve the discoloration of SDF/KI is to cover the lesion with restorative material. However, the risk of discoloration is still considered. 7

Previous investigations focused on the influence of glass ionomer restoration on covering the color of SDF. 5 However, no study has been conducted to assess the effect of delayed application of composite restoration on concealing the color of SDF/KI and its effect on bonding strength. Placing SDF/KI in the cavity after partial caries removal acts as an inhibitor for residual carious progression; however, adding SDF/KI to dentin may affect the bond strength of composite restoration. 8

Our primary objective of this investigation was to evaluate microtensile bond strength and mode of failure of delayed application of composite restoration (self-etch adhesive mode) to artificial carious dentin with and without SDF and KI in primary and permanent teeth.

Our secondary objective was to evaluate the difference in shade between the composite on nontreated dentin and the composite on SDF/KI-treated dentin.

## Materials and Methods

Sample size was determined using the G power statistical power analysis program (version 3.1.9.4) for sample size determination; a total sample size of *n* = 48 (subdivided to 24 in each group; further subdivided to 12 in each subgroup) will be sufficient to detect a large effect size ( *d* ) = 1.06, with an actual power (1– *β* error) of 0.9 (95%) and a significance level of ( *α* error) 0.05 (5%) for two-sided hypothesis test. 9

Forty-eight human teeth were utilized in the study; 24 freshly erupted sound premolar teeth were extracted in the pedodontics department for orthodontic purposes as part of a treatment plan. The pedodontics department provided 24 noncarious deciduous molars, which were extracted as part of a treatment plan. The ethical committee gave its approval for the study's use of human teeth, which were distributed at random among four experimental groups ( *n* = 12). The clearance reference was from the ethical committee, and they followed their guidelines.

### Variables

G1: composite (self-etch adhesive mode) restoration of primary teeth without use of SDF/KI on artificial carious dentin
G2: composite (self-etch adhesive mode) restoration of primary teeth with SDF/KI applied on artificial carious dentin
G3: composite (self-etch adhesive mode) restorations for permanent teeth without the use of SDF/KI on artificial carious dentin
G4: composite (self-etch adhesive mode) permanent teeth restored with SDF/KI on artificial carious dentin

### Specimen Preparation

The teeth were properly cleaned with running water to remove blood. The teeth were scaled to remove calculus and periodontal ligament remains, then polished with fine pumice and soft rubber cups at a standard speed. A magnifying lens was used to examine the teeth for fissures. The teeth with evidence of caries, microcracks, or other faulty structures were eliminated. All samples have been kept in deionized (DI) water containing 0.02% thymol immediately after extraction and used in this study within 1 month.

Each tooth was prepared by removing the roots below the cementoenamel junction using a diamond disc with a high-speed handpiece and DI water irrigation. The remaining coronal tooth structure was vertically impeded in self-curing acrylic resin blocks (Acrostone Dental Factor, England). Acrylic blocks were made with a specially designed cylindrical, split Teflon mold for retaining teeth (4 cm vertically and horizontally). A low-speed diamond blade was used to remove 3 mm of tooth structure from the cusp tip to the middle of the tooth structure, exposing a consistent layer of midcoronal dentin.

### Formation of Artificial-Like Caries Lesion

A coat of varnish was applied to all surfaces of the specimen except the occlusal surface. After that, the collected specimens were submerged for 4 days at 25°C in a demineralizing solution (pH 4.4, 50 mm acetate, 2.2 mm KH2PO4, and 2.2 mm CaCl2). 8

### For Control Group (Nontreated Dentin)

In G1 and G3, DI water was applied to prepared tooth surfaces using a microbrush applicator for 10 seconds and air-dried for 5 seconds. Exposed dentin surfaces were immediately kept in artificial saliva (pH 7.4) containing 5 mM HEPES, 2.5 mM CaCl2•H2O, 0.05 mM ZnCl2, and 0.3 mM NaN3 at 37°C for 14 days. 7

### Application of SDF/KI

In G2 and G4, a drop of SDF solution (Advantage Arrest, Elevate Oral Care) was dispensed into a disposable plastic dish, and a microbrush applicator was utilized to apply SDF to prepared tooth surfaces. The material was then transferred to the dentin surface and actively rubbed for 10 seconds.

Each specimen was thoroughly dried with an air syringe for 5 seconds; the material was left untouched for 1 minute to be absorbed by the tooth. The excess substance was removed with a cotton Q-tip. Using the triple syringe, the specimen was washed with water for 15 seconds. 6

KI (Riva Star, SDI; Bayswater, Australia) was immediately applied for 1 minute till the creamy white became transparent. Distilled water was used to clean all affected surfaces properly. Following SDF/KI treatments per the manufacturer's recommendations, exposed dentin surfaces were immediately kept in artificial saliva (pH 7.4) containing 5 mM HEPES, 2.5 mM CaCl2•H2O, 0.05 mM ZnCl2, and 0.3 mM NaN3 at 37°C for 14 days. 7 10

### Bonding Procedures and Resin Composite Application

The bonding system (3M Scotch bond Universal adhesive) was used for four groups in the self-etch technique (Scotch bond Universal (SBU)). One drop of universal bonding agent was placed on the dentin surface as an active adhesive for 20 seconds. Gentle solvent evaporation with an air syringe was applied for 10 seconds to provide a uniform thin layer. Three successful adhesive layers were softly blasted thin using oil-free compressed air for 10 seconds to provide a homogenous coating of material. Light curing (Elipar DeepCure-S LED Curing Light, 1200 mW/cm ^2^ ) was administered for 10 seconds. 10

To standardize the bonding area, a 6 × 6 mm in diameter and 4 mm height plastic cylindrical-shaped mold was placed on the flat dentin surface in each group. The ring was filled with A3 composite resin from Filtek bulk-fill posterior (3M ESPE, St. Paul, Minnesota, United States). A polyester strip was applied to the top of the filled mold.

The composite restoration was made to a thickness of 4 mm to provide support and attachment to the jig. A glass slip was put over the celluloid strip to create a flat surface and remove the remaining composite resin. The samples were then light-cured from the occlusal surface for 40 seconds using the light-emitting diode light cure system from Dentsply Caulk (Milford, Delaware, United States).

Using a slow-speed handpiece in dry conditions, all specimens' occlusal surfaces and sides were polished utilizing fine, superfine (24 μm), and superfine (8 μm) aluminum oxide polishing disks (Sof-lex, 3M ESPE).

### Evaluation of Shade Matching

The spectrophotometer (VITA Easyshade V, VITA Zahnfabrik, Bad Sackingen, Germany) was used to measure the color of each composite restoration. The following formula was used to determine the shade difference (Δ *E* ) between the restoration and the tooth:

Δ *E* = [(Δ *L* ∗)2 + (Δ *a* ∗)2 + (Δ *b* ∗)2]1/2

where *L* * denotes the color's lightness, *a* *, *b* *, and *c* * stand for the color's redness, greenness, and yellowness, respectively. 11

### Microtensile Bond Strength Test

**Fig. 1:**
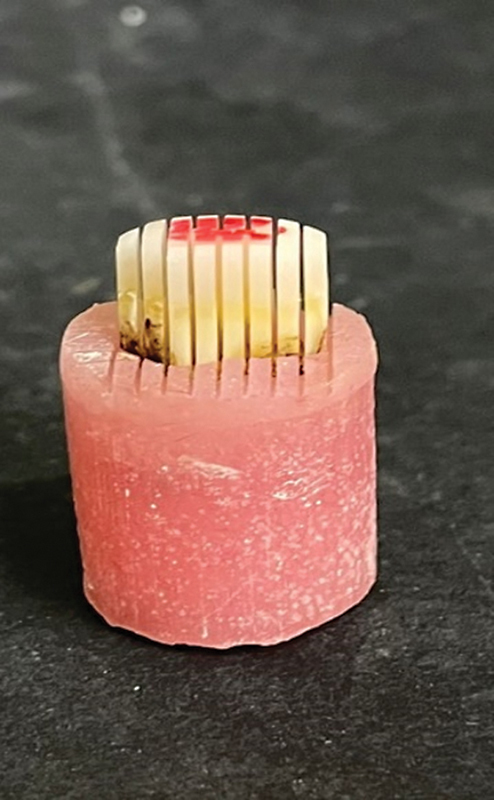
Microtensile bond strength beams selected from coronal parts.

Forty-eight beams were yielded from each restored specimen. Each beam's bonded surface area was measured using a digital caliper. The ends of the adhesive-dentin bonded beams were attached to a jig through cyanoacrylate adhesive and tested under tension using a universal testing machine (Model 2519-104; Instron, United States). The force in Newton (N) required to displace the restoration was measured. The microtensile bond strength (µTBS) was calculated by dividing the load at failure (N) by the cross-sectional bonding area (1 mm ^2^ ). The readings are expressed in mega Pascal (MPa) 12 13 ( Fig. 1 ).

### Failure Mode Analysis

**Fig. 2:**
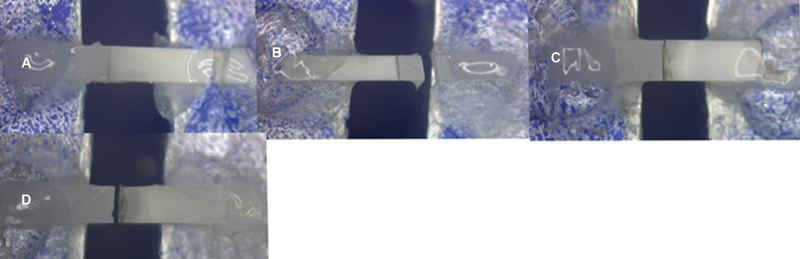
Representative images for the failure mode analysis ( **A** ) Cohesive fracture in dentin. ( **B** ) Cohesive fracture in composite. ( **C** ) Adhesive failure in primary dentition. ( **D** ) Mixed failure in permanent dentition.

After undergoing microtensile testing, the fracture patterns of all the debonded specimens were examined using a stereo microscope at a magnification of 40 × . Four groups were created based on the nature and location of the failure modes:

Type 1: adhesive fracture between adhesive agent and dentin (adhesive failure)
Type 2: adhesive fracture between dentin and adhesive agent plus partial cohesive fracture in the composite restoration or dentin (mixed fracture)
Type 3: cohesive fracture in dentin
Type 4: cohesive fracture in the composite restoration ( Fig. 2 )

### Statistical Analysis

Data management and statistical analysis were carried out with the Statistical Package for Social Sciences (SPSS) version 20. The one-way analysis of variance (ANOVA) test was used to compare groups based on normally distributed numeric variables, followed by Bonferroni's post hoc test for pairwise comparisons. A two-way ANOVA test was employed to investigate the influence of factors and their interaction. The chi-squared test was designed to analyze qualitative data provided as counts and percentages. Each *p* -value is two-sided. *p* -Values of < 0.05 were considered significant.

## Result

**Fig. 3:**
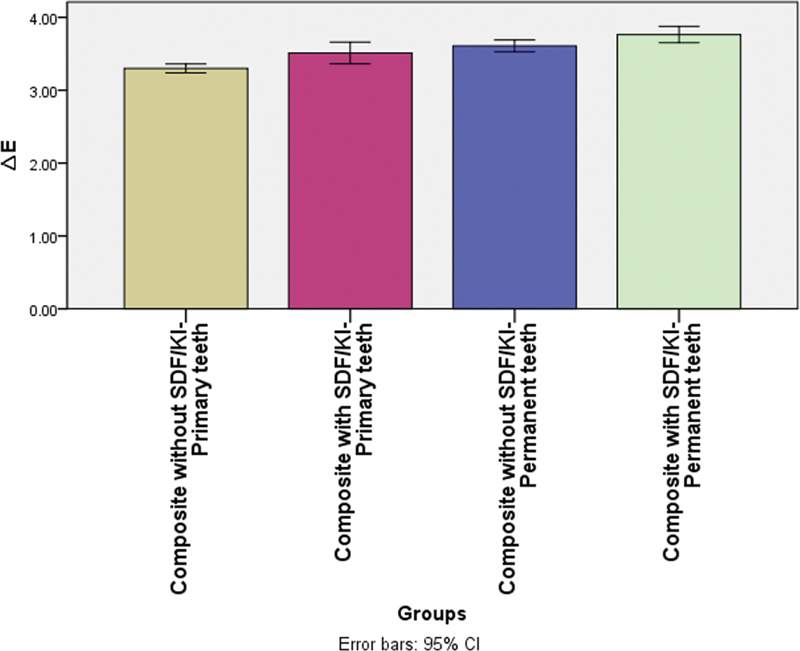
Mean chart illustrating the different modes of failure in different groups.

(1) Regarding color measurement:

Table 1 and Fig. 3 represent the data of the different shades between the groups. The highest mean value was recorded in G4 (composite with SDF/KI, permanent teeth group) (3.763 ± 0.177), followed by G3 (composite without SDF/KI, permanent teeth group) (3.608 ± 0.13), then G2 (composite with SDF/KI, primary teeth group) (3.512 ± 0.236), with the least mean value recorded in G1 (composite without SDF/KI, primary teeth group) (3.299 ± 0.097). The difference between groups was statistically significant ( *p* = 0.000). The post hoc test revealed that G3 was not significantly different from G2 and G4.

**Table 1:** Descriptive statistics and comparison between groups regarding shade difference [∆ *E* ] (ANOVA test)

Groups	Mean	Standard deviation	Standard error	95% confidence interval for mean		Min	Max	*F* -value	*p* -Value
				Lower bound	Upper bound				
G1	3.299 ^c^	0.097	0.028	3.24	3.36	3.15	3.53	16.08	0.000 ^**d**^
G2	3.512 ^b^	0.236	0.068	3.36	3.66	3.24	3.91		
G3	3.608 ^a,b^	0.130	0.037	3.53	3.69	3.46	3.92		
G4	3.763 ^a^	0.177	0.051	3.65	3.88	3.45	3.98		

Abbreviation: ANOVA, analysis of variance.

Note: Significance level *p* ≤ 0.05. Post hoc test: Within the same comparison means with different superscript letter are significantly different.

(2) Microtensile stress at maximum load (MPa);

Table 2 provides a summary of the findings. In deciduous teeth, the mean value in G1 was 23.96 ± 4.09, whereas G2 was 23.83 ± 7.16. The change was not statistically significant ( *p* = 0.941). In permanent teeth, the mean value in G3 was 37.97 ± 5, whereas in G4 was 37.97 ± 5.29. The change was not statistically significant ( *p* = 0.988).

**Table 2:** Descriptive statistics and comparison of microtensile stress at maximum load (MPa) (one-way ANOVA test)

	Mean	Standard deviation	95% confidence interval for mean		Min	Max	*p* -Value within the same dentition	*p* -Value between all subgroups
			Lower bound	Upper bound				
**G1**	** 23.96 ^b^**	**4.09**	**22.27**	**25.64**	**18.76**	**32.43**	**0.941 ns**	
**G2**	** 23.83 ^b^**	**7.16**	**20.88**	**26.79**	**19.43**	**46.34**		0.000*
**G3**	** 37.97 ^a^**	**5.00**	**35.91**	**40.03**	**32.10**	**49.15**	**0.988 ns**	
**G4**	** 37.97 ^a^**	**5.29**	**35.79**	**40.15**	**32.10**	**47.35**		

Abbreviations: ANOVA, analysis of variance; ns, non significant.

Note: Significance level *p* ≤ 0.05, sharing the same superscript letter are not significantly different.

**Fig. 4:**
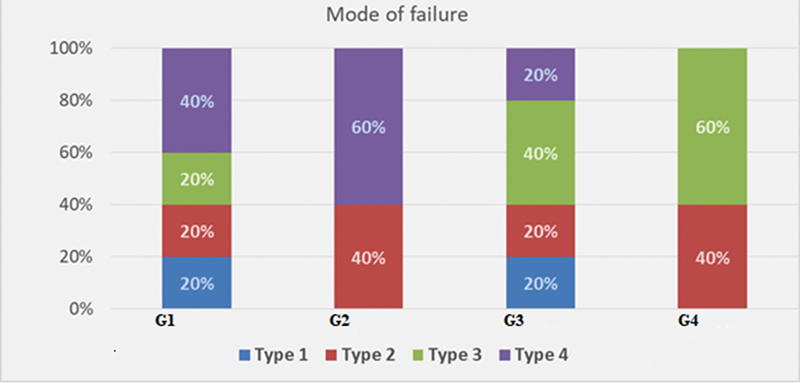
Mean chart illustrating the different modes of failure in different groups.

* Significant, post hoc test.

(3) Mode of failure:

The results are summarized in Fig. 4 .

In deciduous dentition, G1 recorded 20% in types 1, 2, and 3, and 40% in type 4, compared to G2's 0, 40, 0, and 60%. A statistically significant difference was detected ( *p* = 0.005).

In permanent dentition, G3 reported 20% in each of types 1, 2, and 4 and 40% in type 3, compared to 0, 40, 60, and 0% in G4. The result showed a statistically significant difference ( *p* = 0.005).

## Discussion

The current study examined the effect of composite restoration color after 2 weeks of different dentin pretreatments (with or without SDF/KI) in primary and permanent dentations.

In our study, we applied SDF/KI on artificial carious dentin. SDF can be very useful in partial caries removal techniques due to its antibacterial activity in disinfecting the cavity and inhibiting carious propagation and recurrent caries. Also, it was concluded that SDF has the ability to remineralize the demineralized carious dentin. 8 14 15

Also, 14 days were the period of storage of the specimen before applying composite restoration to examine the delayed application of composite on SDF-treated dentin and its effect on bond strength and color of the restoration. 16 17

Moreover, the self-etch adhesive mode was selected in this study due to its mild acidity to avoid the removal of silver ions in SDF with strong acid as in the etch rinse mode. Also, the presence of 10-Methacryloyloxydecyl dihydrogen phosphate (MDP) enhances the bond's strength and durability. 18

The VITA, easy shade spectrophotometer, was used to analyze color because it uses low light intensity to provide great data consistency and strong repeatability for the whole visible spectrum of the laboratory system. 19

The Δ *E* value of composite with SDF/KI indicates its ability to mask color, as there is no significant difference between the control and SDF/KI-treated groups. However, the primary teeth groups were less able to cover color than the permanent teeth groups, which may be explained by the shorter clinical crown of primary teeth compared to permanent teeth, which decreases the ability to mask the color of conditioning. 20

The filler inside the composite, which improves color reflection and covers the color of SDF/KI, may be the primary reason for the good masking effect ability observed in the comparison of conditions between the composite restorations applied in this study after SDF/KI and without SDF/KI. 11

The second reason is that KI reacts with excess silver ions to generate silver iodide, which is easily removed with water and has a yellowish-cream hue, which reverses the staining effect of SDF. 21 22

Moreover, the exposure to the light-curing system in the composite restoration group minimized the creation of the dark metallic silver ions. The results of the present investigation were consistent with those of Raafat et al, who suggested that zirconia-reinforced glass ionomer may effectively cover SDF-induced discoloration. 21

### Regarding Microtensile Bond Strength

The primary goal of our current study was to evaluate the microtensile bond strength and mode of failure of composite on two carious distinct dentitions with and without SDF/KI-treated dentin.

There was no significant difference in delayed self-etch bonding after 14 days with SDF/KI treatment versus untreated artificial carious dentin groups in both dentitions.

There are several possible explanations for this. The first explanation is that using a saturated KI solution could improve dentin bonding. Several investigations revealed that KI could react with residual silver ions and improve the bonding. 23

Another explanation is that delayed self-etches bonding can restore bond strengths, resulting in values comparable to those of untreated dentin. That may be due to delayed bonding having improved Ag stability of SDF particles and remineralization of weak demineralized dentin, which has significant consequences on bonding efficiency. Also, artificial saliva gradually washed away alkaline compounds during storage, lowering the negative buffering effect on functional acidic monomers. So, delayed bonding procedures may be considered an approach to minimize the adverse impacts of SDF/KI treatment. 7 10 24

Several researches have supported our findings, demonstrating that SDF does not affect bond strength while increasing dentin microhardness. It has the potential to reduce ion exchange during acid-base interactions. 23 24 However, Knight et al's investigation showed that leaving SDF/KI precipitates on the dentin surface significantly lowered bonding strength. 6

In this study, permanent teeth demonstrated greater microtensile bond strength than deciduous teeth. Composites applied to permanent teeth have a greater μTBS compared to primary teeth. That could be attributed to dentin's mineral content, as lower concentrations of calcium and phosphorus were measured for primary teeth than permanent teeth. 25

### Regarding the Mode of Failure

Cohesive failure in resin composites exhibited the greatest failure score in the SDF/KI-treated permanent dentin group; however, adhesive failure was absent from this group. Nevertheless, considering that the adhesive layer is still intact, this suggests effective bonding.

Additionally, cohesive failure in dentin demonstrated the greatest failure score in the SDF/KI-treated deciduous dentin group; however, adhesive failure was absent from this group. The bond between the composite and the demineralized dentin was not weakened by the SDF/KI application. 26 These findings coincided with the result of microtensile bond strength.

Our current study limitation is that it was performed *in vitro* using extracted teeth without stimulating the oral condition. Also, the dryness of extracted teeth after the loss of pulp fluid pressure may collapse collagen fibers, decreasing the bond strength, which may affect the accuracy of the result.

## Conclusion

Within the limitation of this study, we conclude that,

1. Composite restoration has a positive effect in covering the color of dentin after the application of the SDF/KI treatments.
2. Postponing the application of composite restoration for 14 days after SDF/KI treatments can improve the bond strength
